# Geospatial analysis of dermatologist distribution and access among US seniors

**DOI:** 10.1016/j.jdin.2024.08.023

**Published:** 2024-10-10

**Authors:** Shawn Afvari, David A. Collet, Anjali Goyal, Esewi Aifuwa, Avery H. LaChance, Bijan Safai

**Affiliations:** a New York Medical College School of Medicine, Valhalla, New York; b Department of Dermatology, Brigham and Women’s Hospital, Boston, Massachusetts; c Department of Dermatology, Metropolitan Hospital Center, New York, New York

**Keywords:** dermatologist shortage, dermatology access, geographic barrier, geographic limitation, health inequity, underserved

*To the Editor:* The United States faces a shortage of dermatologists with a pronounced disparity in their distribution, favoring urban over rural areas.^1^^,^^2^ Patients residing in regions with limited dermatological resources face a heightened risk of advanced skin cancers and poor health outcomes.^3^ This cross-sectional study utilizes geospatial mapping to assess access to dermatological care among Medicare patients across the United States.

Data on all Medicare-participating dermatologists in the United States were acquired from the publicly available 2020 Medicare File, including dermatologist names and practice addresses. Utilizing ARCGIS, a geospatial analysis tool, a real-time map of the United States was generated, marking locations of all dermatology practices serving Medicare patient populations. This map was then superimposed with data from the 2020 Census Bureau File, providing addresses of individuals 65 and older. We calculated the ratios of dermatologists to the population over 65 within their 100-mile radius and created heat maps to illustrate the per capita density of dermatologists. Then, setting a 50-mile and 100-mile radius around each dermatology office on the map, populations residing beyond this radius were identified as geographically challenged. We then compiled lists of areas with the highest patient density that lacked geographical access to dermatological care.

A heat map displaying the per capita density of dermatologists to patients over 65 is shown in Fig 1. A total of 92,240 seniors from 482 zip codes live over 100 miles from a dermatologist (Fig 2), while 451,726 seniors from 2601 zip codes live over 50 miles away. Among locations with the highest concentration of underserved residents, Roswell, New Mexico exhibited the greatest density of individuals, with 7720 residing more than 100 miles from the nearest dermatologist. Texas faced similar challenges in cities such as Del Rio and Eagle Pass, with 5558 and 4667 at-risk individuals, respectively. In the Eastern United States, Maine stood out as the state that presented with the lowest density of dermatologists. A comprehensive list of municipalities and their populations over 65 outside of 100-mile and 50-mile radii is displayed in (Supplementary Tables I and II, available via Mendeley at https://data.mendeley.com/datasets/g53dj58588/1), respectively.

**Fig 1:**
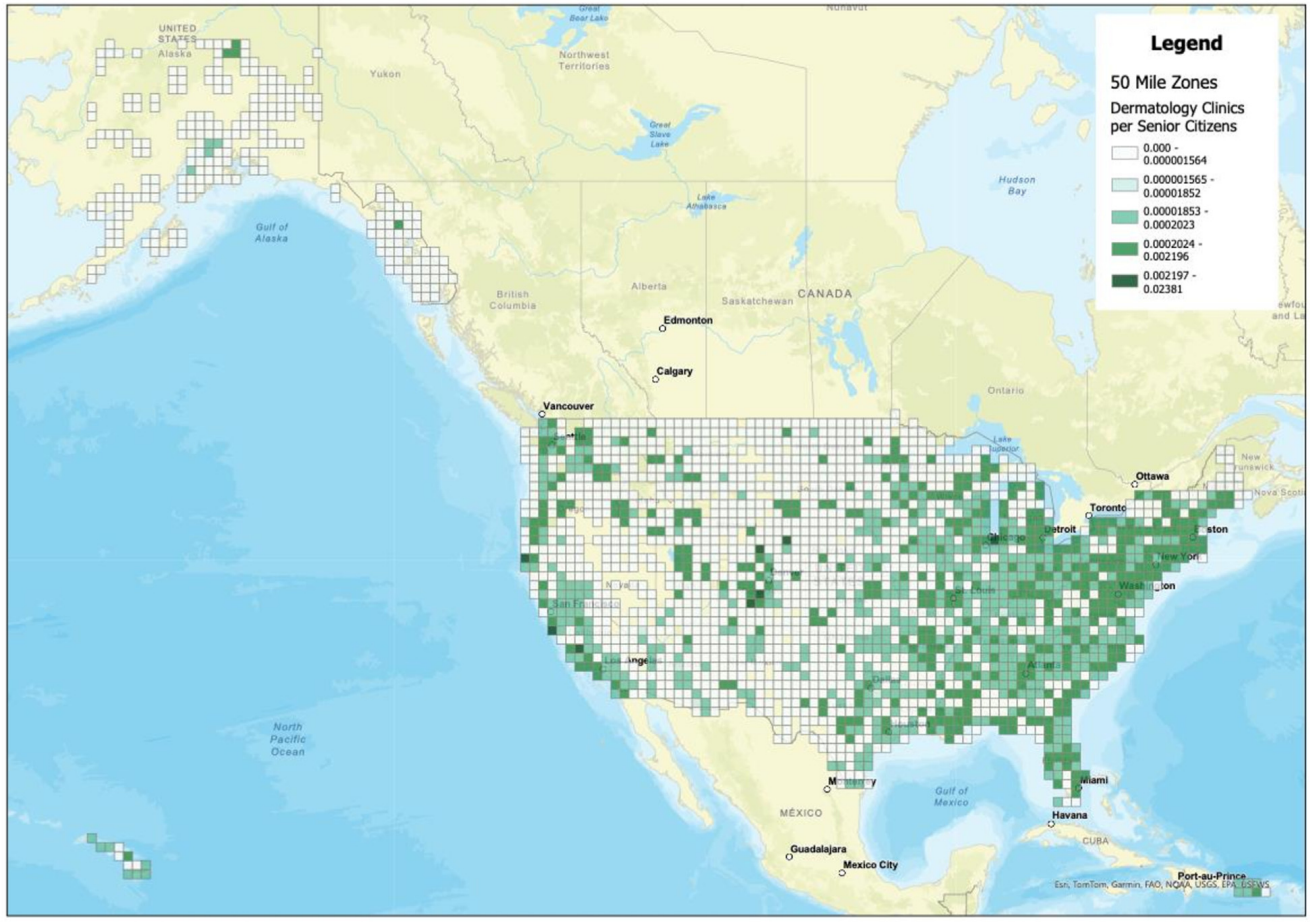
Heat map displaying the per capita density of Medicare-participating dermatologists to populations over 65 years of age.

**Fig 2:**
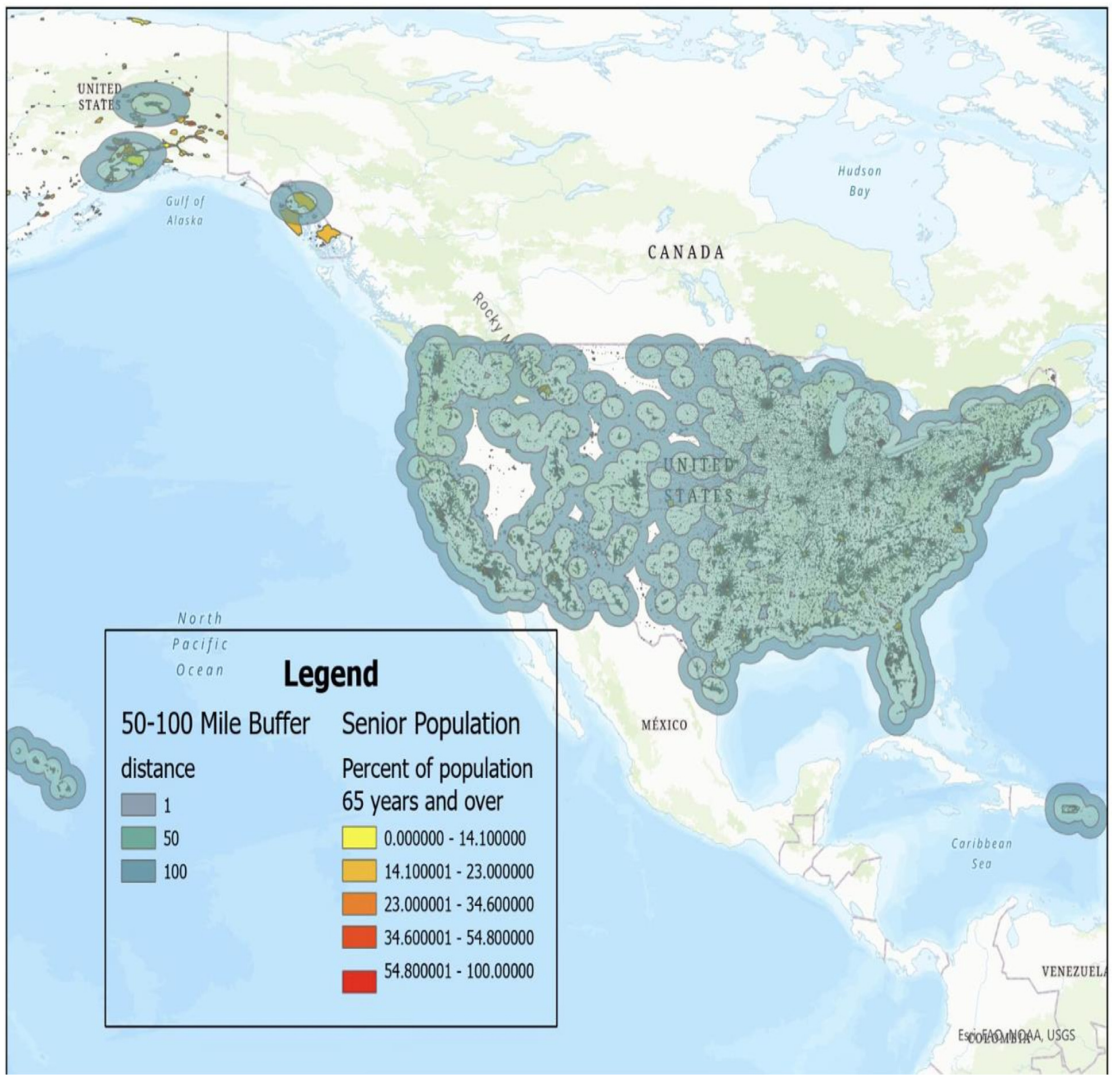
Geographic areas and senior population distribution (65 years and over) beyond 50 to 100 mile buffers surrounding dermatology practices in the United States.

Our analysis identifies specific areas with limited access to dermatological health care. These areas are predominantly situated in southwestern United States and Alaska, reflecting a regional clustering of underserved populations. However, significant involvement of Wyoming and Maine demonstrate the widespread nature of this issue. Our investigation relies upon Medicare data, which limits our analysis to patients 65 and older. However, when we expand our scope to include patients of all ages, we identify a total of 574,249 and 2,435,109 Americans residing 100 miles and 50 miles or more, respectively from their nearest dermatologist. It is important to note that our results reflect a conservative estimate, as a smaller buffer may reveal an even larger population of underserved patients.

This analysis is limited by its focus on geographic access, while other factors, such as long wait times, also play a role in limiting patient access to dermatological care. Another limitation of this study is the exclusion of dermatologists within these regions who may serve patients with private insurance. However, given that only 5.3% of senior populations rely on private insurance alone, we anticipate that non-Medicare participating practices do not majorly bridge the unmet need.^4^ This study highlights specific areas suffering from geographic disparities in dermatological health care, offering essential insights for dermatologists and policymakers, including those within the American Academy of Dermatology, to consider as we strive to enhance access to dermatological care for all patients, regardless of location.

## Conflicts of interest

Author Afvari provides consulting services to Unity Biotechnology. Dr LaChance is the PI for a research study from Pfizer exploring the role of the JAK/STAT pathway in connective tissue disease and a co-I on a research study from Merck exploring the profibrotic niche in systemic sclerosis. Authors Collet, Goyal, and Aifuwa and Dr Safai have no conflicts of interest to declare.
